# Elevated lipoprotein (a) levels are associated with the acute myocardial infarction in patients with normal low-density lipoprotein cholesterol levels

**DOI:** 10.1042/BSR20182096

**Published:** 2019-04-05

**Authors:** Gaojun Cai, Zhiying Huang, Bifeng Zhang, Lei Yu, Li Li

**Affiliations:** 1 Department of Cardiology, Wujin Hospital affiliated with Jiangsu University, Changzhou, Jiangsu 213016, China; 2 Department of Pediatrics, No. 2 Hospital of Changzhou, Changzhou, Jiangsu 213001, China; 3 Postgraduate Medicine, McMaster University, Hamilton, Ontario L8S4L8, Canada

**Keywords:** acute myocardial infarction, coronary artery disease, dyslipidemia, lipid, lipoprotein (a), low density lipoprotein cholesterol

## Abstract

Elevated lipoprotein (a) [Lp(a)] and coronary artery disease (CAD) risk has been renewed interested in recent years. However, the association between Lp(a) and acute myocardial infarction (AMI) risk in patients with normal low-density lipoprotein cholesterol (LDL-C) levels has yet to been established. A hospital-based observational study including 558 AMI patients and 1959 controls was conducted. Lp(a) level was significantly higher in AMI patients with normal LDL-C levels than that in non-CAD group (median: 134.5 mg/l *vs* 108 mg/l, *P*<0.001). According to Lp(a) quartiles (Q1, <51 mg/l; Q2, 51–108 mg/l; Q3, 108–215 mg/l; Q4, ≥215 mg/l), the incidence of AMI increased with the elevated Lp(a) quartiles (*P*<0.001 and *P* for trend<0.001). Logistic regression analysis suggested that patients with Q3 and Q4 of Lp(a) values had 1.666 (95%CI = 1.230–2.257, *P*<0.001) and 1.769 (95%CI = 1.305–2.398, *P*< 0.001) folds of AMI risk compared with patients with Q1, after adjusting for traditional confounders. Subgroup analyses stratified by gender and age showed that the association only existed in male and late-onset subgroups. In addition, we analyzed the association of Lp(a) with AMI risk in different cut-off values (cut-off 1 = 170 mg/l, cut-off 2 = 300 mg/l). A total of 873 (34.68%) and 432 (17.16%) participants were measured to have higher Lp(a) levels according to cut-off 1 and cut-off 2, respectively. Participants with high Lp(a) levels had 1.418- (cut-off1, 95%CI = 1.150–1.748, *P*<0.001) and 1.521- (cut-off 2, 95%CI = 1.179–1.963, *P*< 0.001) folds of AMI risk compared with patients with low Lp(a) levels. The present large-scale study revealed that elevated Lp(a) levels were associated with increased AMI risk in Chinese population with normal LDL-C levels.

## Introduction

Acute myocardial infarction (AMI), including ST segment elevated AMI and non-ST segment elevated AMI, is the most severe type of coronary artery disease (CAD), which results in the disability and sudden death due to the occlusion of coronary arteries. The morbidity and mortality of AMI are increasing worldwide [1]. Low-density lipoprotein cholesterol (LDL-C) is known to play a crucial role in the occurrence and development of CAD, and therapeutics for decreasing LDL-C levels can reduce the risk of major adverse cardiovascular events (MACEs) in patients with CAD. However, some patients who have achieved the guideline recommended LDL-C levels may have high residual cardiovascular risk [2]. Thus, the residual cardiovascular risk has been extensively explored by researchers and clinicians in recent years.

Lipoprotein (a) [Lp(a)], firstly described in 1963 by Berg K, is a LDL-like particle consisting of one apolipoprotein B100 (ApoB100) linked covalently to one molecule of apolipoprotein a [apo(a)] via a single disulfide bond [3]. Apo(a) is a hydrophilic glycoprotein secreted by hepatocyte, which is highly homologous to plasminogen. Differed from plasminogen, apo(a) only has multiple repeats of KIV and one KV domains. Lp(a) concentrations remain stable throughout one’s life, which have a family genetic predisposition and are less affected by diet, environment factors or some lipid-lowing drugs [4]. The Lp(a) KIV-2 copy number mainly affects the concentration of Lp(a). There is a negative correlation between Lp(a) level and KIV-2 copy number. The fewer KIV-2 copy number is associated with the higher Lp(a) concentration [5].

During the last decade, there has been renewed interest in association of Lp(a) and CAD. Lp(a) may promote the occurrence of atherosclerosis by pro-thrombotic, pro-inflammatory and pro-atherogenic effects. Although the physiological functions of Lp(a) are unclear and the mechanism in CAD has not been fully elucidated, epidemiological, Mendelian randomization and genome wide association studies have provided conclusive evidence for the elevated Lp(a) concentration as a causal and independent risk factor of CAD [6–8]. In 2018, Sun et al. recruited 1980 Chinese untreated participants undergoing coronary angiography (CAG) to explore the association between elevated Lp(a) level and the presence and severity of CAD [9]. The levels of Lp(a) were 121.02 (58.75–279.67), 110.59 (56.60–254.40) and 129.95 (60.60–295.70) (mg/l) in total, non-CAD and CAD populations, respectively. They found that elevated Lp(a) was a useful marker to predict CAD and its severity. Only few studies have shown an association between Lp(a) and AMI [10–13], and most of them recruited only small number of patients [11–13]. Moreover, until now, no studies have been carried out to explore the relationship between Lp(a) and AMI risk in patients with normal LDL-C levels. Therefore, the aim of the present study is to determine whether the elevated Lp(a) is associated with the risk of AMI in a Chinese population with normal LDL-C levels.

## Methods

### Study population

The study population was described previously [14]. In brief, subjects who suspected to CAD and underwent CAG between January 2006 and December 2015, were consecutively recruited at Department of Cardiology, Wujin Hospital affiliated to Jiangsu University. Patients with AMI and normal LDL-C levels were enrolled in the present study.

The exclusion criteria were as follows: (1) patient with severe renal or hepatic dysfunction; (2) patient with incomplete data for traditional lipid profiles or Lp(a); (3) AMI not resulting from the atherosclerotic plaque, such as coronary artery spasm, embolism, myocardial bridge, etc. Patients taking lipid-lowering drugs in 3 months prior to the study, which might affect the lipid metabolism, were also excluded from the study [9].

Clinical data and traditional cardiovascular risk factors were collected from patient medical records. The present study complied with the Declaration of Helsinki and was approved by the Ethics Committee of Wujin Hospital. Because the data were retrospectively collected from the electric record, written informed consent was not obtained from the participants.

### Diagnostic criteria

AMI was defined as patients with: (1) typical symptoms of angina more than 30 min; (2) typical rise and gradual fall of biochemical markers of myocardial necrosis (troponin, CM-MB); (3) development of pathologic Q waves; (4) ST segment elevation or depression [15]. All of the patients underwent a CAG examination, which was performed using Judkin’s technique via the radial or femoral artery. Angiograms were analyzed by two experienced cardiologists. At least the vessel stenosis in one of the major coronary arteries was more than 50%. According to coronary angiogram, AMI patients were classified into single-vessel, two-vessel, and three-vessel disease groups.

Non-CAD subjects were defined as those lacking typical angina pectoris symptoms and with the diameter stenosis less than 50% in major coronary arteries. Smoking was defined as regular cigarette smoking. The definitions of essential hypertension (EH) and diabetes mellitus (DM) were described in our previous studies [14]. Late-onset CAD is defined as age older than 60 years in both genders. Normal LDL-C level is defined as the LDL-C level lower than 3.4 mmol/l [16].

### Biochemical parameter analysis

Venous blood was extracted from each participant after 12 h of fasting, and was used to measure total cholesterol (TC), triglyceride (TG), high-density lipoprotein cholesterol (HDL-C), LDL-C, apoprotein A (ApoA), apoprotein B (ApoB), Lp(a), fasting plasma glucose (FPG) and uric acid by automatic biochemical analyzer (Olympus AU5400). The methods to measure biochemical parameters were performed as previously described [17].

### Statistical analysis

The statistical software package SPSS for Windows 17.0 (SPSS Inc., Chicago, IL, USA) was used for the statistical analysis. Continuous data with normal distribution were presented as mean ± standard deviation (SD) and compared by using independent *t* or ANOVA test; Because the distribution of serum Lp(a) and TG was significantly skewed, they were represented as median [quartile ranges (QR)] and were compared by using Mann–Whitney *U* or Kruskal–Wallis *H* test among groups. The normality of the data distribution was assessed with the Kolmogorov–Smirnov test. Categorical variables were expressed as the frequencies and percentages and compared by using Chi-square test. According to the quartiles of Lp(a) values in non-CAD subjects, participants were divided into four subgroups (Q1, <51 mg/l; Q2, 51–108 mg/l; Q3, 108–215 mg/l; Q4, ≥215 mg/l). Multivariable logistic regression, expressed as odds ratio (OR) with 95% confidence interval (95% CI), was used to determine associations between Lp(a) and AMI for Lp(a) quartiles, first for the whole population, then for sub-populations stratified by age, gender, DM and EH status. An initial model 1 was unadjusted for any confounding factors, a subsequent model 2 was adjusted for age, gender, smoking, EH and DM, and a final model 3 was adjusted for factors included in model 2 in addition to TC, HDL-C, LDL-C and ApoA. The correlations between Lp(a) and other lipid profiles were explored by using Spearman analysis (for data that was not normally distributed). A two-side *P* value less than 0.05 was considered to be statistical significant.

## Results

### Baseline characteristics of AMI patients and non-CAD participants

In total, 2517 participants including 558 AMI (452 males and 106 females; mean age, 62.85± 10.22 years) and 1959 non-CAD (1027 males and 932 females; mean age, 60.42 ± 9.52 years), were enrolled in the study. As shown in Table 1, AMI patients were older than non-CAD subjects. Compared with non-CAD individuals, AMI patients had higher prevalence of male, smoking, EH and DM. In addition, the levels of Lp(a), HDL-C and ApoA were significantly lower in the AMI patients than those in the non-CAD participants. On the other hand, Lp(a) levels in AMI group were significantly higher than those in non-CAD group. There were no difference in TG, LDL-C, ApoB and non-HDL-C levels between AMI group and non-CAD group.

**Table 1:** Baseline characteristics of AMI patients and non-CAD participants

Characteristics	Total (*n* = 2517)	Non-CAD (*n* = 1959)	AMI (*n* = 558)	*P*
Age, years	60.96 ± 9.73	60.42 ± 9.52	62.85 ± 10.22	**<0.001**
Male, *n* (%)	1479 (58.76)	1027 (52.42)	452 (81.00)	**<0.001**
Smoking, *n* (%)	596 (23.68)	367 (18.73)	229 (41.04)	**<0.001**
EH, *n* (%)	1487 (59.08)	1129 (57.63)	358 (64.16)	**0.006**
DM, *n* (%)	372 (14.78)	242 (12.35)	130 (23.30)	**<0.001**
TC, mmol/l	4. 37 ± 0.77	4.39 ± 0.78	4.28 ± 0.74	**0.002**
TG, mmol/l	1.38 (0.98–2.04)	1.37 (0.98–2.05)	1.40 (1.00–2.03)	0.764
HDL-C, mmol/l	1.14 ± 0.32	1.15 ± 0.32	1.07 ± 0.30	**<0.001**
LDL-C, mmol/l	2.49 ± 0.53	2.48 ± 0.53	2.53 ± 0.53	0.052
ApoA, g/l	1.16 ± 0.24	1.17 ± 0.24	1.10 ± 0.22	**<0.001**
ApoB, g/l	0.82 ± 0.24	0.82 ± 0.23	0.83 ± 0.25	0.172
Non-HDL-C, mmol/l	3.23 ± 0.73	3.24 ± 0.72	3.21 ± 0.74	0.414
Lp(a), mg/l	113 (55–228.5)	108 (51–215)	134.5 (73–258)	**<0.001**
Lp(a)≥170 mg/l, *n* (%)	873 (34.68)	644 (32.87)	229 (41.04)	**<0.001**
Lp(a)≥300 mg/l, *n* (%)	432 (17.16)	312 (15.93)	120 (21.51)	**0.003**

Abbreviations: AMI, acute myocardial infarction; Apo, apolipoprotein; CAD, coronary artery disease; DM, diabetes mellitus; EH, essential hypertension; HDL-C, high-density lipoprotein cholesterol; LDL-C, low-density lipoprotein cholesterol; TC, total cholesterol; TG, triglyceride.

Bold values indicate statistical significance.

### Clinical characteristics of participants according to Lp(a) quartiles

Table 2 lists the clinical characteristics of individuals according to Lp(a) quartiles. Compared with those in Q1 group, participants in Q2 to Q4 group were older. There were significant trends for the gradual increase in age, TC, HDL-C and LDL-C levels according to Lp(a) quartiles (age, *P* = 0.016 and *P* for trend = 0.009; TC, *P*<0.001 and *P* for trend<0.001; HDL-C, *P*<0.001 and *P* for trend<0.001; LDL-C, *P*<0.001 and *P* for trend<0.001). On the contrary, TG level was gradually decreased with the elevation of Lp(a) quartiles (*P*<0.001 and *P* for trend<0.001). The non-HDL-C levels in Q4 groups were significantly higher than those in Q2 group (*P* = 0.008). However no significant increases were observed among four groups (*P* for trend = 0.071).

**Table 2:** Clinical characteristics of individuals according to Lp(a) quartiles

Characteristics	Q1 (<51mg/l)	Q2 (51–108mg/l)	Q3 (108–215 mg/l)	Q4 (≥215 mg/l)	*P*	*P_trend_*
Age, years	60.01 ± 9.92	61.26 ± 9.55	60.67 ± 9.74	61.79 ± 9.65	**0.009**	**0.006**
Male, *n* (%)	327 (56.48)	360 (58.73)	398 (60.39)	394 (59.16)	0.569	0.286
Smoking, *n* (%)	129 (22.28)	140 (22.84)	167 (25.34)	160 (24.02)	0.589	0.319
EH, *n* (%)	357 (61.66)	353 (57.59)	370 (56.15)	407 (61.11)	0.130	0.793
DM, *n* (%)	99 (17.10)	89 (14.52)	89 (13.51)	95 (14.26)	0.321	0.147
AMI, *n* (%)	95 (16.41)	121 (19.74)	167 (25.34)	175 (26.28)	**<0.001**	**<0.001**
TC, mmol/l	4.34 ± 0.81	4.29 ± 0.80	4.35 ± 0.72	4.47 ± 0.74	**<0.001**	**0.001**
TG, mmol/l	1.56 (1.08–2.57)	1.42 (0.98–2.04)	1.32 (0.95–1.91)	1.28 (0.95–1.81)	**<0.001**	**<0.001**
HDL-C, mmol/l	1.11 ± 0.32	1.11 ± 0.31	1.14 ± 0.30	1.18 ± 0.33	**<0.001**	**<0.001**
LDL-C, mmol/l	2.39 ± 0.57	2.46 ± 0.54	2.52 ± 0.51	2.59 ± 0.49	**<0.001**	**<0.001**
ApoA, g/l	1.18 ± 0.25	1.15 ± 0.23	1.14 ± 0.23	1.16 ± 0.24	0.024	0.097
ApoB, g/l	0.83 ± 0.27	0.81 ± 0.24	0.81 ± 0.21	0.83 ± 0.23	0.150	0.886
Non-HDL-C, mmol/l	3.23 ± 0.78	3.18 ± 0.76	3.21 ± 0.68	3.29 ± 0.69	**0.031**	**0.071**
Lp(a), mg/l	30 (20–40)	78 (64–90)	147 (125–179)	354 (268–491)	**<0.001**	**<0.001**

Abbreviations: AMI, acute myocardial infarction; Apo, apolipoprotein; DM, diabetes mellitus; EH, essential hypertension; HDL-C, high-density lipoprotein cholesterol; LDL-C, low-density lipoprotein cholesterol; Q, quartile; TC, total cholesterol; TG, triglyceride.

Bold values indicate statistical significance.

### Associations of Lp(a) with the risk of AMI

Lp(a) level was significantly higher in AMI patients with normal LDL-C levels than that in non-CAD group (median: 134.5 mg/l *vs* 108 mg/l, *P*<0.001) (Table 1). Then, we explored the relationship between Lp(a) and the severity of AMI according to coronary angiogram. We found that Lp(a) level was not associated with the number of coronary artery lesion (*P* = 0.173) (Supplementary Table S1).

**Figure 1:**
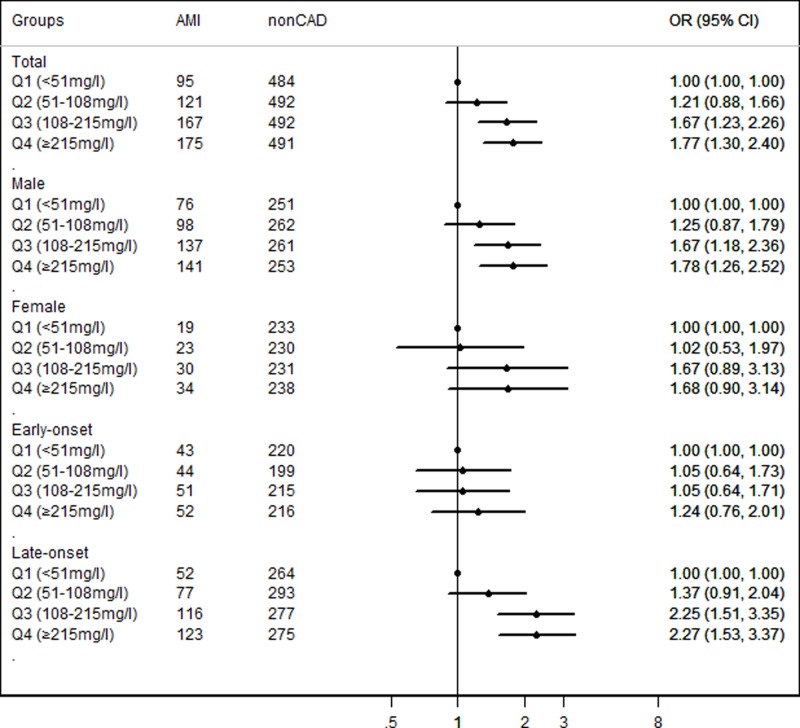
Association between Lp(a) quartiles and AMI risk in whole population, age and gender subgroups Adjustment for age, gender, smoking, EH, DM, TC, HDL-C, LDL-C and ApoA.

According to Lp(a) quartiles, the prevalence of AMI was elevated with the increase in Lp(a) quartiles (*P*<0.001 and *P* for trend<0.001) (Table 2). Furthermore, logistic regression analysis was carried out to explore the relationship between Lp(a) quartiles and the risk of AMI. As shown in Figure 1, Lp(a) were significantly associated with AMI risk. After adjusted for traditional confounders, patients with Q3 and Q4 of Lp(a) values had 1.666 (95%CI = 1.230–2.257, *P*<0.001) and 1.769 (95%CI = 1.305–2.398, *P*< 0.001) folds of AMI risk comparing with patients with Q1, respectively (Supplementary Table S2).

Furthermore, subgroup analyses stratified by gender and age showed that the association only existed in male (for Q3, adjusted OR = 1.673, 95% CI = 1.184–2.364, *P* = 0.004; for Q4, adjusted OR = 1.779, 95% CI = 1.256–2.520, *P* = 0.001) and late-onset (for Q3, adjusted OR = 2.249, 95% CI = 1.511–3.348, *P*<0.001; for Q4, adjusted OR = 2.266, 95% CI = 1.526–3.365, *P*<0.001) subgroups (Figure 1, Supplementary Table S2). Subgroup analyses based on the EH and DM status revealed that elevated Lp(a) was associated with AMI risk in both EH and non-EH subgroups. However, there were no difference in the association between Lp(a) value and AMI risk in DM subgroup (Supplementary Figure S1 and Supplementary Tables S3).

### Baseline characteristics of participants in different cut-off value group

Table 3 lists the baseline characteristics of participants in low and high Lp(a) level groups according to different cut-off values (cut-off 1 = 170 mg/l; cut-off 2 = 300 mg/l). Participants with higher Lp(a) levels groups had higher TC, HDL-C and LDL-C levels compared with lower Lp(a) levels groups. However, TG levels in higher Lp(a) levels groups were significantly lower compared with lower Lp(a) levels groups (cut-off 1, 1.45 mmol/l *vs* 1.25 mmol/l, *P*<0.001; cut-off 2, 1.40 mmol/l *vs* 1.29 mmol/l, *P*<0.05). In addition, age and prevalence of smoking in higher Lp(a) level groups were significantly higher than those in lower Lp(a) levels groups.

**Table 3:** Baseline characteristics of participants in different cut-off value groups

Characteristics	Cut-off1		Cut-off 2	
	<170 mg/l (*n* = 1644)	≥170 mg/l (*n* = 873)	<300 mg/l (*n* = 2085)	≥300 mg/l (*n* = 432)
Age, year	60.64 ± 9.74	61.56 ± 9.68*	60.83 ± 9.73	61.56 ± 9.69
Male, *n* (%)	953 (57.97)	526 (60.25)	1226 (58.80)	253 (58.56)
Smoking, (%)	182 (11.07)	214 (24.51)***	493 (23.65)	103 (23.84)
EH, %	967 (58.82)	520 (59.56)	1224 (58.71)	263 (60.88)
DM, %	251 (15.27)	121 (13.86)	306 (14.68)	66 (15.28)
AMI, %	329 (20.01)	229 (26.23)***	438 (21.01)	120 (27.78)***
TC, mmol/l	4.32 ± 0.78	4.45 ± 0.74***	4.34 ± 0.78	4.48 ± 0.74**
TG, mmol/l	1.45 (1.0–2.19)	1.25 (0.93–1.80)***	1.40 (0.9–2.09)	1.29 (0.96–1.83)*
HDL-C, mmol/l	1.12 ± 0.31	1.17 + 0.32***	1.12 ± 0.32	1.19 ± 0.31***
LDL-C, mmol/l	2.45 ± 0.55	2.58 + 0.49***	2.47 ± 0.54	2.59 ± 0.49*
ApoA, g/l	1.16 ± 0.24	1.15 ± 0.24	1.15 ± 0.24	1.17 ± 0.23
ApoB, g/l	0.82 ± 0.25	0.82 ± 0.22	0.82 ± 0.24	0.84 ± 0.24
LP (a), mg/l	72 (39–1110)	294 (219–442)***	90.0 (48–152)	445 (360–650)***

Abbreviations: AMI, acute myocardial infarction; Apo, apolipoprotein; DM, diabetes mellitus; EH, essential hypertension; HDL-C, high-density lipoprotein cholesterol; LDL-C, low-density lipoprotein cholesterol; TC, total cholesterol; TG, triglyceride.

Bold values indicate statistical significance.

*Compared with low Lp(a) group, *P*<0.05; **compared with low Lp(a) group, *P*<0.01; ***compared with low Lp(a) group, *P*<0.001.

### Association of Lp(a) with AMI risk in different cut-off values

**Figure 2:**
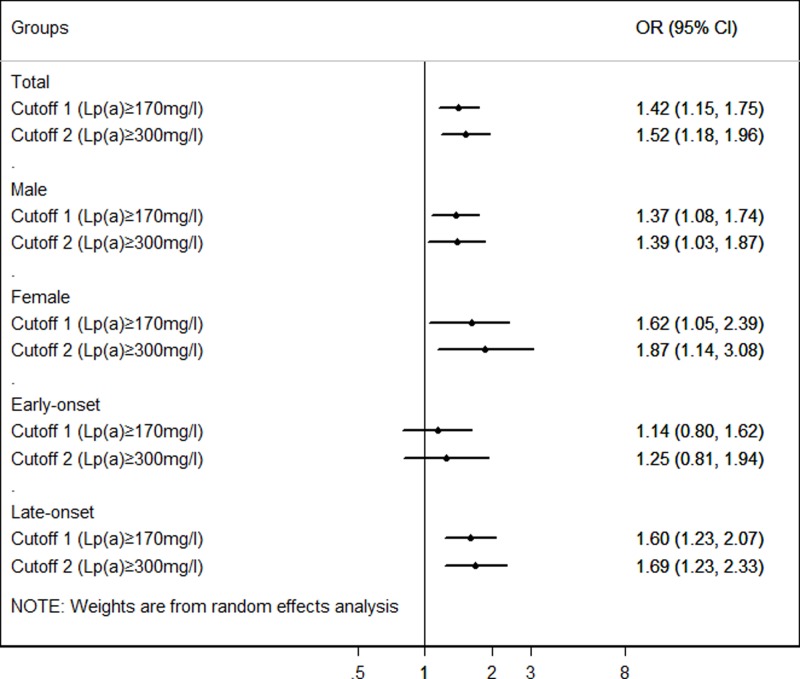
Association between Lp(a) and AMI risk in different cut-off values in whole population, age and gender subgroups Adjustment for age, gender, smoking, EH, DM, TC, HDL-C, LDL-C and ApoA.

We also analyzed the association of Lp(a) with AMI risk in different cut-off values. As shown in Table 1, the prevalence of Lp(a) ≥ 170 mg/l and Lp(a) ≥ 300 mg/l in the whole population were 34.68% and 17.16%, respectively. In comparison with non-CAD participants, AMI patients had higher prevalence of Lp(a) ≥ 170 mg/l (26.23% *vs* 20.01%, *P*<0.001) and Lp(a) ≥ 300 mg/l (27.78% *vs* 21.01%, *P*<0.001). Furthermore, logistic regression analysis was carried out to explore the relationship between Lp(a) quartiles and the risk of AMI in different cut-off values (Figure 2). Participants with high Lp(a) levels had 1.418- (cut-off1, 95%CI = 1.150-1.748, *P*<0.001) and 1.521- (cut-off 2, 95%CI = 1.179–1.963, *P*< 0.001) folds of AMI risk compared with patients with low Lp(a) levels, after adjusting for traditional confounders (Supplementary Table S4).

### Correlation between Lp(a) levels and other lipid profiles

After adjustment for age, gender, smoking, EH and DM status, Lp(a) levels were positively associated with TC, HDL-C, LDL-C and ApoB levels (*P* all <0.05), and negatively associated with TG levels (*P*<0.001) (Table 4).

**Table 4:** Line logistic regression of association of LP (a) with traditional risk factor of AMI

	TC	TG	HDL-C	LDL-C	ApoA	ApoB
*r*	0.075	−0.075	0.073	0.131	0.011	0.045
P^*^	**<0.001**	**<0.001**	**<0.001**	**<0.001**	0.284	**0.013**

Abbreviations: AMI, acute myocardial infarction; Apo, apolipoprotein; HDL-C, high-density lipoprotein cholesterol; LDL-C, low-density lipoprotein cholesterol; TC, total cholesterol; TG, triglyceride.

* Adjustment for age, gender, smoking, EH and DM.

## Discussion

Our data showed that Lp(a) values were significantly associated with AMI risk in participants with normal LDL-C levels, and the highest quartiles of Lp(a) had 1.769-folds of AMI risk comparing with participants with the lowest quartiles.

More than 90% of variation of Lp(a) concentrations is explained by apo (a) gene polymorphism [18]. Because the Lp(a) concentrations remain stable throughout one’s life, it is accepted that measurement of Lp(a) levels only once in a person’s lifetime is adequate for his or her CAD risk prediction. However, the distribution of Lp(a) was skewed significantly, and the levels of Lp(a) varied greatly in different populations, which ranged from <0.1 mg/dl to more than 200 mg/dl. In the present hospital-based study, a total of 2517 participants with normal LDL-C levels, involving 558 AMI patients and 1959 non-CAD subjects were enrolled. The median of Lp(a) was 113 mg/l in whole population, 108 mg/l in non-CAD and 134.5 mg/l in AMI groups, which was consistent with the result from Sun et al.’s Study [5], higher than that in Cui FM’s study (median value 56 mg/l) [19], but significantly lower than that in other ethnic populations [20,21]. The discrepancy might partly be explained by several causes. Firstly, the different selected population characteristics (such as the prevalence of age, gender, ethnicity, diagnostic criteria of disease and controls, etc.) and different research designs (cohort, case–control and cross-sectional study) might be an important cause. The present study was a hospital-based observational study, and all of participants came from Changzhou, Wujin district. In our study, the diagnoses of AMI and non-CAD were verified by CAG examination and all of the participants did not take lipid-lowering drugs in the three months prior to the study, which might be lack of the effect of lipid-lowering drugs on the Lp(a) concentrations. Additionally, different LPA gene polymorphism across ethnicities leading to different Lp(a) concentrations might be another reason [22]. Furthermore, different methods of measurement also lead to the discrepancy of results [23].

Numerous studies have indicated that elevated Lp(a) was also associated with cardiovascular risk factors, such as EH, DM, metabolic syndrome, and aortic valve stenosis [24–28]. In 2016, Afshar et al. [29] conducted a case-only study to identify interactions between Lp(a) and other risk factors for CAD. They found that high Lp(a) interacted with LDL-C level, but the interaction becomes attenuated at LDL-C ≤3.5 mmol/l. In recent year, the role of Lp(a) in the occurrence and development of CAD has been widely investigated. Elevated Lp(a) levels are identified as a casual risk factor for CAD, besides traditional risk factors, such as diabetes, hypertension and age [6,30,31]. For example, Cai et al. identified that the median value of Lp(a) was 74 mg/l in 6125 non-CAD participants and 101 mg/l in 636 prior CAD patients [6]. Either on continuous or categorical scales, the risk for CAD was significantly stepwise increased with the increased Lp(a) levels or quintiles, and was modified by some risk factors. In addition, elevated plasma Lp(a) levels were associated with increased risk of cardiovascular events [32]. In 2018, a prospective study involved 1602 Chinese patients with stable CAD to evaluate the association between Lp(a) and the risk of MACEs. The baseline Lp(a) levels were 134 (70–276) mg/l in Chinese stable CAD patients. The mean follow-up period was 39.6 months. After adjustment for traditional cardiovascular risk factors, the hazard ratio for MACEs was 1.291 per standardized deviation in the log-transformed Lp(a) level with 95% CI (1.091–1.527). Moreover, Lp(a) lowering therapy could decrease the incidence of PCI or surgical myocardial revascularization in patients with high Lp(a) values and angiographically documented CAD [33].

Epidemiological studies showed that an upward trend in AMI mortality in China [1], despite the development of emergency percutaneous coronary intervention in recent years. The unstability of the coronary artery atherosclerotic plaque was the pathophysiological basis of AMI. Lp(a) might take a role in the initiation and development of AMI through inducing the chemotactic activity to peripheral monocytes, attenuating fibrinolysis and promoting coagulation [26]. In recent years, studies had been reported the association of Lp(a) with the risk factor for AMI [20]. In the Chronic Renal Insufficiency Cohort (CRIC) study, the authors found that the highest quartile of Lp(a) was associated with an increase in AMI, death and composite outcome after 7.5 year follow-up, and concluded that Lp(a) was an independent risk factor of AMI and death [20]. However, only few studies have shown an association of Lp(a) with AMI in Chinese. In the present study, the results firstly showed that Lp(a) level was significantly higher in AMI patients with normal LDL-C levels than that in non-CAD group. According to Lp(a) quartiles, the prevalence of AMI was elevated with the increase in Lp(a) quartiles (*P*<0.001 and *P* for trend<0.001). Logistic regression analysis also suggested that patients with Q3 and Q4 of Lp(a) values had 1.666- (95%CI = 1.230–2.257, *P*<0.001) and 1.769- (95%CI = 1.305–2.398, *P*<0.001) folds of AMI risk comparing with patients with Q1, after adjusting for traditional confounders. Clinically, physicians treat patients with high LDL-C levels, while Lp(a) research sheds light on a new serum marker, which has yet to be considered in cardiovascular risk management. The clinical relevance provided by the current study is that more attention should be paid to elevated Lp(a) levels in participants with normal LDL-C levels.

Furthermore, stratified analysis based on age and gender suggested that the association of Lp(a) with AMI risk only existed in male and late-onset subgroups, which was consistent with Mahabadi AA’s study [28,34], but inconsistent with Rallidis LS’s results [35]. Interestingly, our results also revealed that elevated Lp(a) was associated with AMI risk in non-DM subgroup, whereas not in DM subgroup.

In 2007, the cut-off value of Lp(a) predicting CAD risk in Chinese population was recommended as 300 mg/l, which was reported in Chinese guidelines on prevention and treatment of dyslipidemia [36]. However, Cui et al. detected the Lp(a) levels in a Chinese health check-up population included 9238 individuals, and proposed Lp(a) less than 170 mg/l as cut-off value according to the relative risk for AMI for Chinese Han ethnicity recently [19]. In the present study, we also attempted to analyze the association of Lp(a) and risk of AMI in different cut-off values. According to different cut-off values, the prevalence of Lp(a) levels ≥170 mg/l was 34.68%, and ≥300 mg/l was 17.16%, respectively. The results showed that Lp(a) levels were associated with the risk of AMI in both cut-off values (cut-off value of 170 mg/l, OR = 1.418, 95%CI = 1.150–1.748, *P* = 0.001; cut-off value of 300 mg/l, OR = 1.521, 95%CI = 1.179–1.963, *P* = 0.001), which suggested that Lp(a) cut-off value of 300 mg/l had higher AMI risk than that of 170 mg/l.

## Limitations

Firstly and most importantly, this is a hospital-based cross-sectional study, which could not identify the causal association between Lp(a) concentrations and risk of AMI in participants with normal LDL-C levels. Multi-center and prospective study design should be carried out. Secondly, the Lp(a) levels in our study were detected as mass concentration but not particle concentration, which might lead to the discrepancy of measurements. Thirdly, some effective data could not be obtained from the medical records because of the retrospective study design, which might have an impact on the results.

## Conclusion

The present large-scale study revealed that elevated Lp(a) levels were associated with increased AMI risk in Chinese population with normal LDL-C levels. In addition, Lp(a) cut-off value of 300 mg/l had higher risk of AMI compared with that of 170 mg/l. Due to the limitation of the design of study, the results should be verified by further prospective, large-scale and multi-center studies.

## Supporting information

**supplementary Figure 1:** 

**Supplemental Table 1.** The relationship between Lp(a) and the severity of AMI

**Supplemental Table 2.** Unadjusted and adjusted multivariable logistic regression of associations of Lp(a) with AMI

**Supplemental Table 3.** Unadjusted and adjusted multivariable logistic regression of associations of Lp(a) with AMI in EH and DM subgroups

**Supplemental Table 4.** Unadjusted and adjusted multivariable logistic regression of associations of Lp(a) with AMI in different cutoff values

## Abbreviations

AMI: acute myocardial infarction
ApoA: apoprotein A
ApoB: apoprotein B
CAG: coronary angiography
CAD: coronary artery disease
CRIC: Chronic Renal Insufficiency Cohort
95% CI: 95% confidence interval
DM: diabetes mellitus
EH: essential hypertension
FPG: fasting plasma glucose
HDL-C: high-density lipoprotein cholesterol
OR: odds ratio
LDL-C: low-density lipoprotein cholesterol
TC: total cholesterol
TG: triglyceride
