# Supplementary material for: Elevated lipoprotein (a) levels are associated with the acute myocardial infarction in patients with normal low-density lipoprotein cholesterol levels
Source: Biosci Rep. 2019 Apr 5;39(4):BSR20182096. doi: 10.1042/BSR20182096 (PMC6449519; doi:10.1042/BSR20182096)
Supplement: Supplementary file 1 [file bsr-39-bsr20182096_Supp1.pdf]

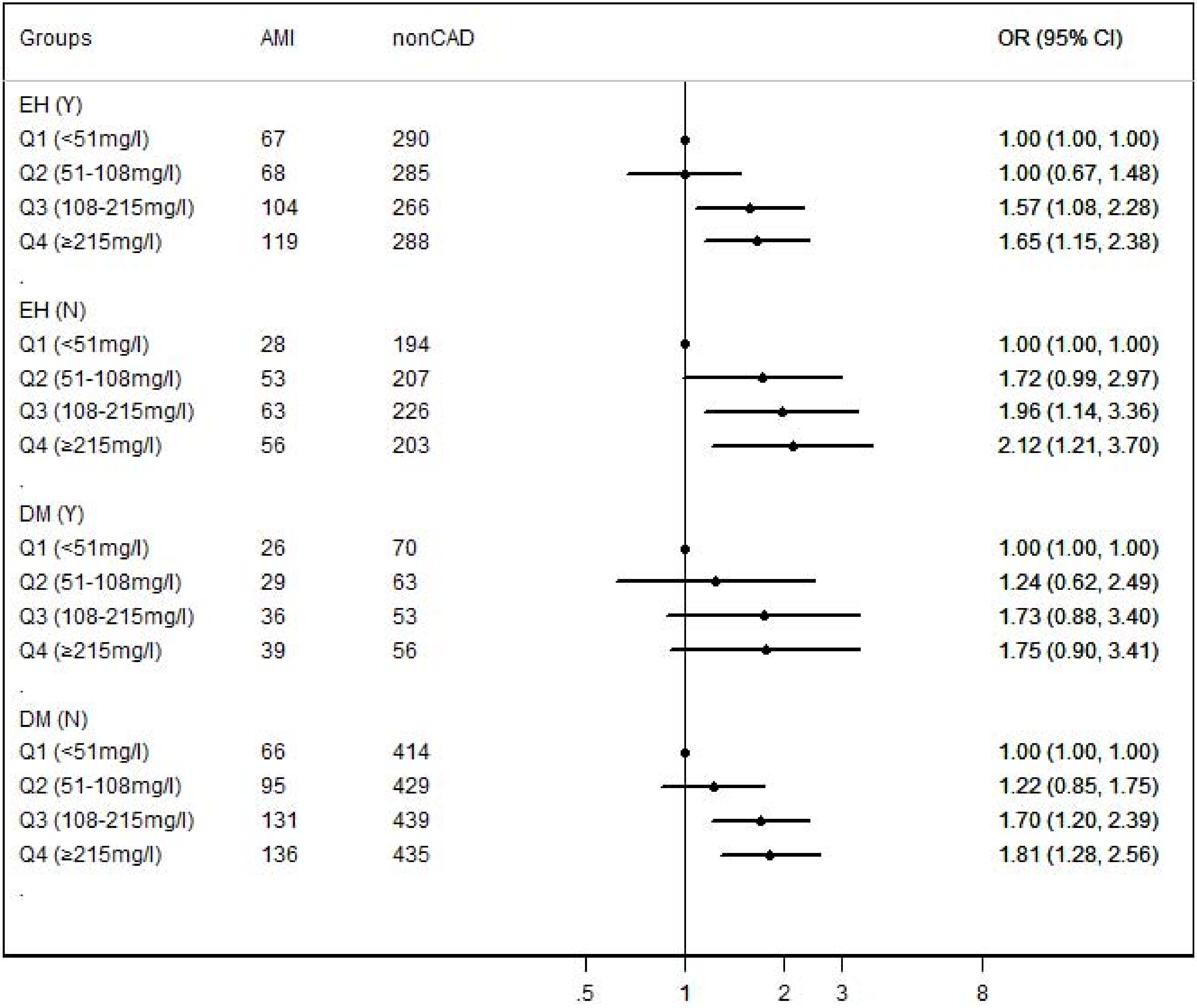
Groups
AMI
nonCAD
OR (95% CI)
EH (Y)
Q1 (<51mg/l) 67 290 1.00 (1.00, 1.00)
Q2 (51-108mg/l) 68 285 1.00 (0.67, 1.48)
Q3 (108-215mg/l) 104 266 1.57 (1.08, 2.28)
Q4 (≥215mg/l) 119 288 1.65 (1.15, 2.38)
EH (N)
Q1 (<51mg/l) 28 194 1.00 (1.00, 1.00)
Q2 (51-108mg/l) 53 207 1.72 (0.99, 2.97)
Q3 (108-215mg/l) 63 226 1.96 (1.14, 3.36)
Q4 (≥215mg/l) 56 203 2.12 (1.21, 3.70)
DM (Y)
Q1 (<51mg/l) 26 70 1.00 (1.00, 1.00)
Q2 (51-108mg/l) 29 63 1.24 (0.62, 2.49)
Q3 (108-215mg/l) 36 53 1.73 (0.88, 3.40)
Q4 (≥215mg/l) 39 56 1.75 (0.90, 3.41)
DM (N)
Q1 (<51mg/l) 66 414 1.00 (1.00, 1.00)
Q2 (51-108mg/l) 95 429 1.22 (0.85, 1.75)
Q3 (108-215mg/l) 131 439 1.70 (1.20, 2.39)
Q4 (≥215mg/l) 136 435 1.81 (1.28, 2.56)
.5
1
2
3
8

Supplemental Table 1. The relationship between Lp(a) and the severity of AMI

Variables	single-vessel (n=229)	two-vessel (n=189)	three-vessel (n=140)	*P*
Age, year	61.07± 10.01	63.86± 10.81	64.38± 9.31	0.002
TC, mmol/l	4.26± 0.73	4.32± 0.74	4.25± 0.77	0.608
TG, mmol/l	1.38 (1.01-2.03)	1.41 (0.98-2.06)	1.42 (1.01-1.89)	0.908
HDL-C, mmol/l	1.09± 0.31	1.06± 0.29	1.04± 0.29	0.313
LDL-C, mmol/l	2.51± 0.54	2.57± 0.48	2.52± 0.56	0.439
Lp(a), mg/l	133 (64.5-255)	128 (79.0-230.5)	148 (80.3-315.8)	0.173

TC, total cholesterol; TG, triglyceride; HDL-C, high-density lipoprotein cholesterol; LDL-C, low-density lipoprotein cholesterol

Supplemental Table 2. Unadjusted and adjusted multivariable logistic regression of associations of Lp(a) with AMI

	Q1		Q2		Q3		Q4	
	OR (95%CI)	*P*	OR (95%CI)	*P*	OR (95%CI)	*P*	OR (95%CI)	*P*
**Total**								
Model 1	1.000 (Ref.)	-	1.253 (0.931-1.685)	0.136	1.729 (1.305-2.291)	**<0.001**	1.816 (1.373-2.402)	**<0.001**
Model 2	1.000 (Ref.)	-	1.290(0.944-1.763)	0.110	1.756(1.303-2.366)	**<0.001**	1.863(1.385-2.506)	**<0.001**
Model 3	1.000 (Ref.)	-	1.213(0.884-1.665)	0.231	1.666(1.230-2.257)	**<0.001**	1.769(1.305-2.398)	**<0.001**
**Gender**								
**Male**								
Model 1	1.000 (Ref.)	-	1.235(0.874-1.746)	0.231	1.734(1.247-2.410)	**0.001**	1.841(1.324-2.558)	**<0.001**
Model 2	1.000 (Ref.)	-	1.315(0.920-1.879)	0.132	1.753(1.248-2.463)	**0.001**	1.853(1.320-2.603)	**<0.001**
Model 3	1.000 (Ref.)	-	1.251(0.872-1.795)	0.224	1.673(1.184-2.364)	**0.004**	1.779(1.256-2.520)	**0.001**
**Female**								
Model 1	1.000 (Ref.)	-	1.226(0.650-2.313)	0.528	1.593(0.872-2.910)	0.130	1.752(0.971-3.160)	0.062
Model 2	1.000 (Ref.)	-	1.130(0.591-2.162)	0.711	1.773(0.955-3.293)	0.070	1.874 (1.022-3.437)	**0.042**
Model 3	1.000 (Ref.)	-	1.019(0.526-1.973)	0.956	1.666(0.888-3.125)	0.112	1.683(0.903-3.137)	0.101
**Age**								
**Early-onset**								
Model 1	1.000 (Ref.)	-	1.131(0.713-1.795)	0.601	1.214(0.776-1.898)	0.396	1.232(0.789-1.923)	0.359
Model 2	1.000 (Ref.)	-	1.115(0.685-1.816)	0.661	1.157(0.718-1.865)	0.550	1.311(0.817-2.104)	0.262
Model 3	1.000 (Ref.)	-	1.054(0.644-1.726)	0.835	1.049(0.645-1.706)	0.847	1.238(0.762-2.013)	0.388
**Late-onset**								
Model 1	1.000 (Ref.)	-	1.334(0.904-1.969)	0.146	2.126(1.472-3.072)	**<0.001**	2.271(1.575-3.273)	**<0.001**
Model 2	1.000 (Ref.)	-	1.453(0.966-2.184)	0.073	2.290(1.552-3.378)	**<0.001**	2.371(1.612-3.486)	**<0.001**
Model 3	1.000 (Ref.)	-	1.370(0.906-2.073)	0.136	2.249(1.511-3.348)	**<0.001**	2.266(1.526-3.365)	**<0.001**

AMI, acute myocardial infarction; Q, quartile; OR, odds ratio; CI, confidence interval.

Model 1, crude model without adjustment for any facors; Model 2, adjustment for age, gender, smoking, EH, DM;

Model, adjustment for factors in model 2 plus TC, HDL-C, LDL-C and ApoA.

Bold values indicate statistical significance.

Supplemental Table 3. Unadjusted and adjusted multivariable logistic regression of associations of Lp(a) with AMI in EH and DM subgroups

	Q1		Q2		Q3		Q4	
	**OR (95%CI)**	***P***	**OR (95%CI)**	***P***	**OR (95%CI)**	***P***	**OR (95%CI)**	***P***
**EH**								
**N**								
Model 1	1.000 (Ref.)	-	1.774(1.078-2.919)	0.024	1.931(1.189-3.136)	**0.008**	1.911(1.166-3.134)	**0.010**
Model 2	1.000 (Ref.)	-	1.709(0.997-2.930)	0.051	1.936(1.140-3.290)	**0.015**	1.994(1.161-3.425)	**0.012**
Model 3	1.000 (Ref.)	-	1.717(0.993-2.969)	0.053	1.962(1.145-3.364)	**0.014**	2.118(1.214-3.697)	**0.008**
**Y**								
Model 1	1.000 (Ref.)	-	1.033(0.710-1.502)	0.866	1.692(1.193-2.400)	**0.003**	1.788(1.272-2.515)	**0.001**
Model 2	1.000 (Ref.)	-	1.086(0.736-1.602)	0.679	1.698(1.180-2.444)	**0.004**	1.817(1.273-2.594)	**0.001**
Model 3	1.000 (Ref.)	-	0.999(0.673-1.483)	0.996	1.571(1.084-2.279)	**0.017**	1.653(1.147-2.381)	**0.007**
**DM**								
**N**								
Model 1	1.000 (Ref.)	-	1.389(0.987-1.955)	0.060	1.872(1.353-2.590)	**<0.001**	1.961(1.419-2.710)	**<0.001**
Model 2	1.000 (Ref.)	-	1.311(0.919-1.870)	0.135	1.814(1.293-2.546)	**0.001**	1.948(1.390-2.728)	**<0.001**
Model 3	1.000 (Ref.)	-	1.220(0.852-1.748)	0.278	1.698(1.204-2.394)	**0.003**	1.811(1.283-2.555)	**0.001**
**Y**								
Model 1	1.000 (Ref.)	-	0.996(0.531-1.869)	0.990	1.640(0.895-3.004)	0.109	1.681(0.927-3.041)	0.087
Model 2	1.000 (Ref.)	-	1.251(0.638-2.453)	0.515	1.599(0.836-3.058)	0.156	1.660(0.876-3.146)	0.120
Model 3	1.000 (Ref.)	-	1.244(0.623-2.486)	0.536	1.730(0.879-3.405)	0.112	1.753(0.902-3.406)	0.098

EH, essential hypertension; DM, diabetes mellitus. N, no; Y, yes; OR, odds ratio; CI, confidence interval. Q, quartile;

Bold values indicate statistical significance.

Supplemental Table 4. Unadjusted and adjusted multivariable logistic regression of associations of Lp(a) with AMI in different cutoff values

	Cut-off 1		Cut-off 2	
	**OR(95%CI)**	***P***	**OR(95%CI)**	***P***
**Total**				
Model 1	1.421(1.172-1.724)	**<0.001**	1.446(1.143-1.830)	**0.002**
Model 2	1.413(1.151-1.735)	**0.001**	1.465(1.139-1.885)	**0.003**
Model 3	1.418(1.150-1.748)	**0.001**	1.521(1.179-1.963)	**0.001**
**Gender**				
**Male**				
Model 1	1.394(1.110-1.751)	**0.004**	1.375(1.035-1.826)	**0.028**
Model 2	1.361(1.076-1.721)	**0.010**	1.341(1.001-1.798)	**0.049**
Model 3	1.370(1.078-1.740)	**0.010**	1.392(1.034-1.872)	**0.029**
**Female**				
Model 1	1.473(0.978-2.220)	0.064	1.857(1.166-2.958)	**0.009**
Model 2	1.643(1.076-2.510)	**0.022**	1.823(1.120-2.967)	**0.016**
Model 3	1.618(1.049-2.393)	**0.029**	1.874(1.142-3.076)	**0.013**
**Age**				
**Early-onset**				
Model 1	1.143(0.823-1.586)	0.426	1.186(0.794-1.771)	0.406
Model 2	1.149(0.810-1.630)	0.435	1.224(0.797-1.881)	0.356
Model 3	1.137(0.797-1.623)	0.479	1.251(0.807-1.940)	0.317
**Late-onset**				
Model 1	1.594(1.252-2029)	**<0.001**	1.631(1.215-2.190)	**0.001**
Model 2	1.586(1.230-2.046)	**<0.001**	1.623(1.184-2.223)	**0.003**
Model 3	1.598(1.232-2.073)	**<0.001**	1.694(1.233-2.327)	**0.001**

AMI, acute myocardial infarction; OR, odds ratio; CI, confidence interval.

Bold values indicate statistical significance.

Model 1, crude model without adjustment any factors; Model 2, adjustment for age, gender, smoking, EH, DM; Model, adjustment for factors in Model 2 plus TC, HDL-C, LDL-C and ApoA.
